# Multimodal digital phenotyping of diet, physical activity, and glycemia in Hispanic/Latino adults with or at risk of type 2 diabetes

**DOI:** 10.1038/s41746-023-00985-7

**Published:** 2024-01-11

**Authors:** Amruta Pai, Rony Santiago, Namino Glantz, Wendy Bevier, Souptik Barua, Ashutosh Sabharwal, David Kerr

**Affiliations:** 1 Electrical and Computer Engineering, Rice University, Houston, TX USA; 2 Sansum Diabetes Research Institute, Santa Barbara, CA USA; 3 Santa Barbara County Education Office, Children & Family Resource Services, Santa Barbara, CA USA; 4 Sutter Center for Health Systems Research, Santa Barbara, CA USA

**Keywords:** Predictive markers, Lifestyle modification

## Abstract

Digital phenotyping refers to characterizing human bio-behavior through wearables, personal devices, and digital health technologies. Digital phenotyping in populations facing a disproportionate burden of type 2 diabetes (T2D) and health disparities continues to lag compared to other populations. Here, we report our study demonstrating the application of multimodal digital phenotyping, i.e., the simultaneous use of CGM, physical activity monitors, and meal tracking in Hispanic/Latino individuals with or at risk of T2D. For 14 days, 36 Hispanic/Latino adults (28 female, 14 with non-insulin treated T2D) wore a continuous glucose monitor (CGM) and a physical activity monitor (Actigraph) while simultaneously logging meals using the MyFitnessPal app. We model meal events and daily digital biomarkers representing diet, physical activity choices, and corresponding glycemic response. We develop a digital biomarker for meal events that differentiates meal events into normal and elevated categories. We examine the contribution of daily digital biomarkers of elevated meal event count and step count on daily time-in-range 54-140 mg/dL (TIR_54–140_) and average glucose. After adjusting for step count, a change in elevated meal event count from zero to two decreases TIR_54–140_ by 4.0% (*p* = 0.003). An increase in 1000 steps in post-meal step count also reduces the meal event glucose response by 641 min mg/dL (*p* = 0.0006) and reduces the odds of an elevated meal event by 55% (*p* < 0.0001). The proposed meal event digital biomarkers may provide an opportunity for non-pharmacologic interventions for Hispanic/Latino adults facing a disproportionate burden of T2D.

## Introduction

Beyond genetic and biological factors, behavioral and sociocultural influences, including diet and physical activity choices, are essential factors in determining the risk of progression of type 2 diabetes (T2D)^1,2^. Wearable devices and mobile health smartphone applications are promising digital data sources for diet, physical activity choices, and glycemic response to support more personalized management of T2D^3,4^. Digital phenotyping refers to continuous quantification of an individual’s bio-behaviors using digital data^5,6^. Quantification requires the development of digital biomarkers that represent objective data-driven physiological and behavioral measures.

An important application of digital phenotyping is actionable insights for patients, e.g., the ability to determine which combination of diet and exercise is beneficial for them. Towards that, current approaches have mainly analyzed continuous glucose data and focused on aggregate measures of glycemic control over longer durations, e.g., time-in-range and glycemic variability over 2 weeks^7^. While the aggregate measures have been demonstrated to have clinical relevance^8,9^, they provide little actionable benefit to patients in making their daily diet and exercise decisions. To provide actionable recommendations, we need multimodal digital phenotyping allowing simultaneous capture of diet, exercise, and glycemic profiles. Additionally, digital biomarkers around specific events (e.g., meals, day-to-day) that provide granularity to understand the causal impact of diet and exercise actions on glycemic response have to be developed.

To the best of our knowledge, there is a lack of studies investigating multimodal digital phenotyping for T2D, let alone in Hispanic/Latino populations. Hispanic/Latino populations face a disproportionate burden of T2D, with the prevalence of both diagnosed and undiagnosed T2D nearly twice as high among the U.S., Mexican-origin Hispanic/Latino adults compared to non-Hispanic Whites^10–12^. Past work has shown that among Hispanic/Latino adults living with or at risk of T2D, continuous glucose monitoring (CGM) can provide digital biomarkers beyond traditional measures of time-in-range, average glucose, and glycemic variability^13,14^. Similarly, measuring the intensity and timing of physical activity, assessed by wearable physical activity trackers, has been demonstrated feasible for this population^15^. While the feasibility of continuous glucose monitors and wearable activity trackers have been demonstrated, simultaneous use of the modalities along with food logging has not been explored in the Hispanic/Latino population. Our primary objectives were: (1) Evaluate the feasibility of multimodal digital data collection from Hispanic/Latino adults with or at risk of T2D, (2) to model meal events and daily digital biomarkers of diet, physical activity, and glycemic response using multimodal digital data and, (3) assess cause–effect relationship between digital biomarkers.

**Fig. 1:**
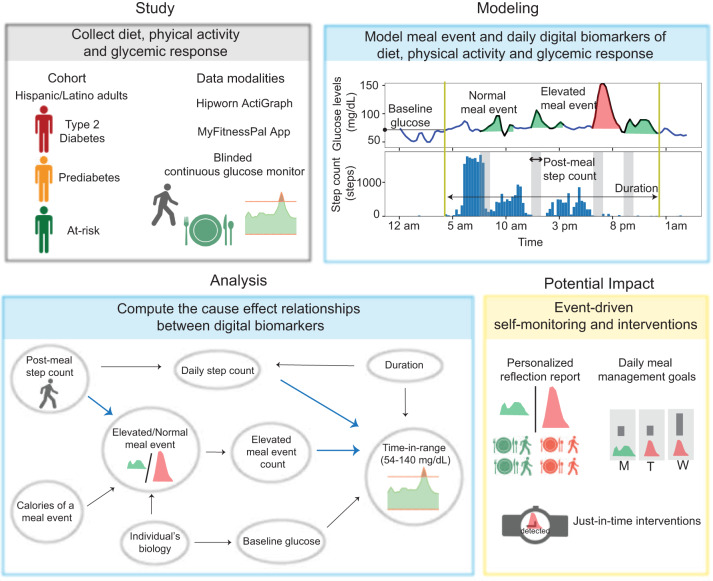
Research summary. Overall contributions of multimodal digital phenotyping in Hispanic/Latino adults with or at risk of non-insulin treated T2D.

Our work demonstrates the application of multimodal digital phenotyping, i.e., the simultaneous use of CGM, physical activity monitors, and meal tracking in Hispanic/Latino individuals with or at risk of T2D. The overall summary of the paper is displayed in Fig. 1. We conducted our study in 36 Hispanic/Latino adults with or at risk of T2D and observed high levels of adherence across all modalities. We compute digital biomarkers for meal events, such as the meal event’s glucose response, post-meal step count, and calorie content of the meal event using self-reported food logs, step count data from the physical activity monitor, and CGM curves. Further, we developed a digital biomarker for meal events that differentiates meal events into normal and elevated categories using CGM and HbA1c data. We also propose digital biomarkers for daily monitoring, such as the elevated meal event count and daily step count. We compute the cause-effect relationships between our proposed daily digital biomarkers and known glycemic outcomes, such as time-in-range and average glucose, while controlling for confounders like baseline glucose at the start of the day, and duration of the day. Our findings indicate that the proposed meal event and daily biomarkers can be specific targets for adaptive lifestyle and non-pharmaceutical interventions to help achieve glycemic goals.

## Results

### Description of cohort and feasibility of study

**Fig. 2:**
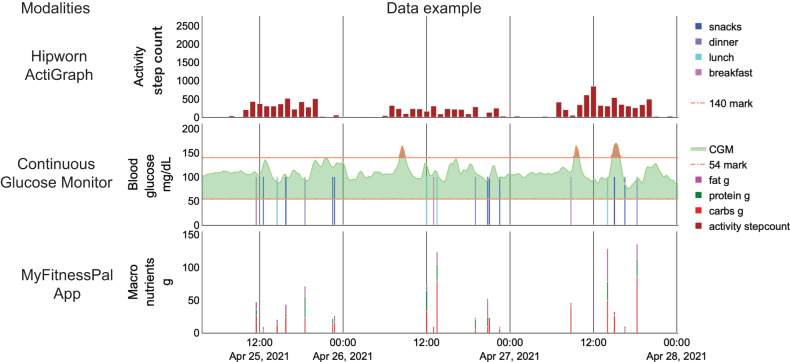
Visualization of a snapshot of the multimodal data. Digital health devices were used to collect synchronous information on the meal, activity, and glucose excursions in free-living conditions.

We conducted a study in a Hispanic/Latino cohort with varying diabetes status (Table 1). Information on occupation is included in Supplementary Table 10. The study described in methods was approved by the Rice University Institutional Review Board (IRB-FY 2021-54). The dataset captured food choices (via MyFitnessPal App), physical activity (via hipworn ActiGraph), and glucose excursions (via CGM) in free-living conditions of 36 Hispanic/Latino participants with or at risk of type 2 diabetes (Fig. 2). Each food log consisted of the timing of the log, macronutrient composition, and meal occasion label. Physical activity measure was the number of steps (step count) taken every minute calculated using the Actilife software from raw accelerometer sensor data. Finally, glucose excursions were measured as interstitial fluid glucose levels every 15 min. The median adherence in days for MyFitnessPal meal logging was 13 [interquartile range (IQR): 9–14]; for CGM, 13 [IQR: 13–13] days; and for ActiGraph, 13 [IQR: 9–14]. The median simultaneous adherence was 11 [IQR: 5–13] days. We observed high adherence for individual modalities. The percentage of participants having at least 10 days of adherence to CGM, MyFitnessPal, and ActiGraph was 94%, 75%, and 75%, respectively. Simultaneous adherence is often difficult to achieve in multimodal studies. Hence, we chose 50% as the threshold, i.e., if more than 50% of the participants had at least 10 days of simultaneous adherence, then using these modalities for data collection is feasible in this population. We found that 52% of our participants displayed at least 10 days of simultaneous adherence to all three modalities. Hence, we conclude that multimodal monitoring in the Hispanic/Latino population is feasible.

**Table 1:** Demographic and clinical measurements for the participant cohort.

Variable (*n* = 36 participants)	Median [interquartile range]
Age (years)	51 [39–59]
Gender	28 female, 8 male
Body mass index (kg/m^2^)	32.6 [29.6–35.2]
HbA_1c_ (%)	6.0 [5.5–6.9]
Number of participants with known diabetes	14 (38%)
Number of participants born in the United States	6 (17%)
Number of participants with no insurance coverage	11 (31%)
Diabetes status	
At-risk	13 (36%)
Prediabetes	9 (25%)
Type 2 Diabetes	14 (39%)

### Meal events and daily digital biomarkers

We used the self-reported meal log timings and CGM curves to identify the start time of meal events. We implemented a framework to correct the meal log timings and add meal timings as self-reported logs are error-prone (described in “Methods”).

**Fig. 3:**
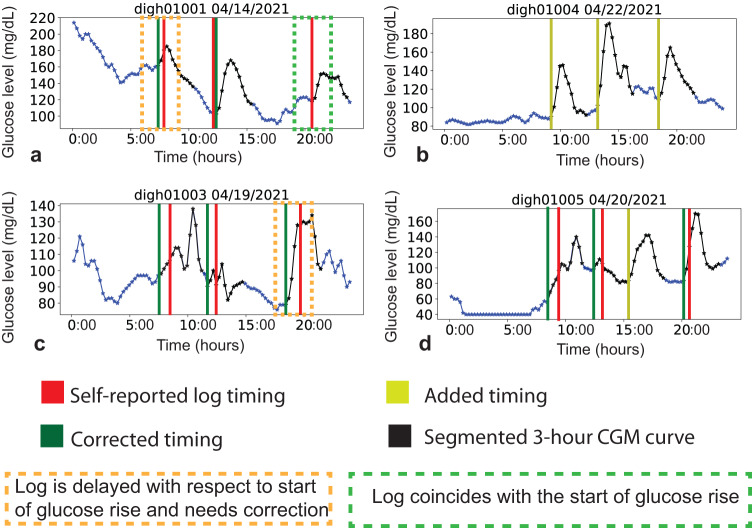
Meal event annotations. Various examples of CGM profiles overlayed with meal timings across different participants are shown in (**a**–**d**). The red vertical lines correspond to self-reported food log timings. The green lines adjacent to the red lines correspond to corrected timing. The yellow lines correspond to added timings.

In total, 1467 self-reported meal logs were provided by participants. For each self-reported log, each annotator independently identified a corrected timing or tagged the log unknown as described in the methods. Further, each annotator independently added timings as discussed in the methods. A few examples of corrected and added timing are shown in Fig. 3. The breakdown of the number of correction and addition annotations for each of the four annotators is presented in Supplementary Table 1. There were a total of 6725 annotated timings combined across all four annotators. We aggregated the annotated timings as described in methods and identified 1584 meal events. To investigate inter-annotator agreement, we analyzed the intra-class correlation of the corrected timings across 951 logs (logs for which all four annotators had been able to identify a corrected timing). The intra-class correlation was found to be 0.95.

Second, we developed digital biomarkers to analyze each meal event, namely calorie content of the meal event, post-meal step count, meal event’s glucose response denoted as ${\rm{MGR}}_{3\,{\rm{h}}}$ (incremental area under the 3-h CGM curve from the start time of the meal event^14,16^), and elevated/normal classification of the meal event. We developed a new CGM-driven meal event characterization that uses an “elevated” or “normal” classification of the meal by exploiting the mapping between ${\rm{MGR}}_{3\,{\rm{h}}}$ and ${\rm{HbA}_{1c}}$ observed across participants (explained in methods).

**Fig. 4:**
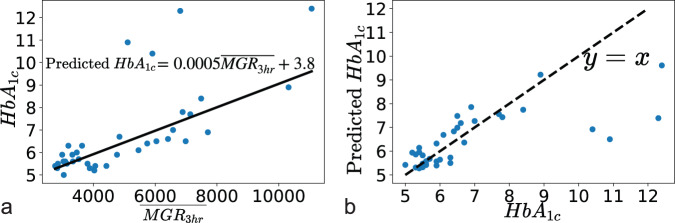
Mapping between HbA_1c_ and average meal event glucose response. The scatterplot on the left **a** displays the robust regression fit between average meal event glucose response and HbA_1c_. The equation representing the best line-of-fit is reported (solid black line). The scatterplot in **b** shows the correlation between true and fitted HbA_1c_ values. The dashed black line represents the 45° line.

We calculated ${\rm{MGR}}_{3\,{\rm{h}}}$ for each of the total 1584 meal events and ${\overline{\rm{MGR}}}_{3\,{\rm{h}}}$ for each of the 36 participants. The line of fit for the robust linear regression model between ${\overline{\rm{MGR}}_{3\,{\rm{h}}}}$ and ${\rm{HbA}_{1c}}$ is shown in Fig. 4a. The association between true and fitted values of ${\rm{HbA}_{1c}}$ is shown in Fig. 4b. The correlation between true and fitted values was significant with a Pearson correlation coefficient of 0.72 (*p* < 0.0001, beta distribution test). The mean absolute error was 0.80%. Error analysis after excluding the four samples of extremely high values of ${\rm{HbA}_{1c}}$ (${\rm{HbA}_{1c}}$ > 10) resulted in a mean absolute error of 0.41% and Pearson’s correlation coefficient of 0.88 (*p* < 0.0001, beta distribution test).

We used the model to characterize individual meal events as elevated or normal (from a glucose perspective). ${\overline{\rm{MGR}}_{3\,{\rm{h}}}}$ quantifies a person’s average glycemic regulation to meals, and its mapping to their baseline HbA_1c_ is provided by the model. The thresholds for elevated/normal classification were based on the ${\rm{HbA}_{1c}}$ definitions for at-risk, prediabetes, and T2D^17^.

**Fig. 5:**
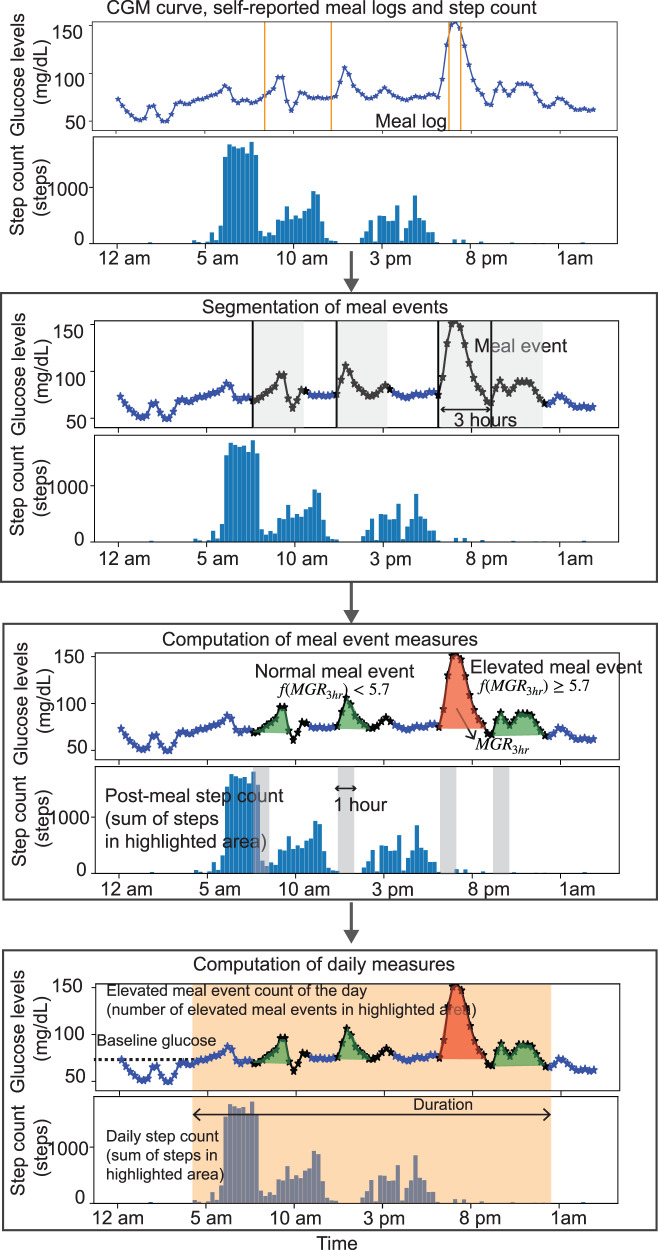
Illustration of steps taken in methods. The CGM curve and meal logs are analyzed to segment meal events. The meal event measures are the meal event’s glucose response (${\rm{MGR}}_{3\,{\rm{h}}}$), post-meal step count, and elevated or normal classification of meal events computed for each meal event. Daily measures of elevated meal count, daily step count, baseline glucose, and duration are then calculated.

Third, we constructed daily digital biomarkers. We defined a duration (analysis window) for each day and computed daily measures of the digital biomarkers, such as daily elevated meal event count that is the number of meal events that were classified as elevated in that duration. We determined the daily *step count* as the total steps taken within the duration. An overview of the steps of computation is shown in Fig. 5. Additionally, we computed baseline glucose at the start time of day.

### Daily elevated meal event count and step count have a significant effect on time-in-range and average glucose

We computed the effect of daily elevated meal event count and daily step count on daily glycemic outcomes to evaluate the significance of the proposed digital biomarkers for monitoring and event-driven recommendations. The outcomes describing glycemic control were time-in-range (54–140 mg/dL) and average glucose was measured using CGM values during the duration. First, we investigated the effect of a day’s elevated meal event count on glycemic outcomes while adjusting for confounders such as baseline glucose at the start of the day, daily step count, and duration of the day (explained in Methods). We had 369 daily samples across 36 participants.

**Fig. 6:**
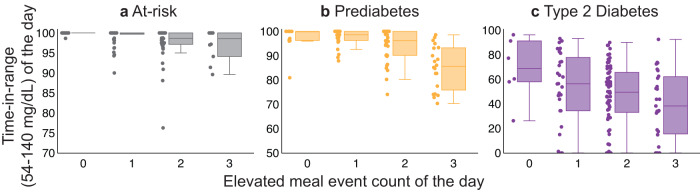
Association between Daily TIR_54__–__140_ and elevated meal event count. Daily TIR_54__–__140_ decreases associated with an increase in elevated meal event count across participants **a** at risk of T2D, **b** with prediabetes, **c** with T2D. The ends of the box represent the lower and upper quartiles. The median (second quartile) is marked by a line inside the box. The data points adjacent to the box plot display the underlying data points where each data point corresponds to a day.

Figure 6 demonstrates a negative association between the elevated meal event count and time-in-range with TIR_54-140_ decreasing with an increase in elevated meal event count across participants who are at risk of or with prediabetes or T2D. The decrease in TIR_54__–__140_ and increase in average glucose due to the increase in the elevated meal event count is summarized in Table 2. An increase in average glucose and a decrease in TIR_54__–__140_ for a change in elevated meal event count from 0 to 1 was not statistically significant. However, the change from 0 to 2 and 0 to 3 was statistically significant.

**Table 2:** Effect of daily step count and elevated meal event count on glycemic outcomes.

Glycemic Variable	Time-in-range (54–140 mg/dL) Percentage of total time (%)	Average glucose mg/dL
Variable of interest	Estimate (standard error, t-test p-value)	Estimate (standard error, *t*-test *p*-value)
Elevated meal event count 0–1	−1.1 (1.3, 0.41)	1.7 (1.2, 0.16)
Elevated meal event count 0–2	−4.0 (1.3, 0.003)	5.8 (1.3, <0.0001)
Elevated meal event count 0 to ≥3	−8.1 (1.5, <0.0001)	7.7 (1.4, <0.0001)
Daily step count (1 unit = 1000 steps)	0.07 (0.1, 0.63)	−0.3 (0.1, 0.02)

Time-in-range (54–140 mg/dL) refers to the percentage of total time glucose values between 54 and 140 mg/dL. The effects of elevated meal event count were computed after adjusting for confounders, namely baseline glucose, duration and daily step count. Similarly, the effects of daily step count were computed after adjusting for confounders’ baseline, glucose, duration, and elevated meal event count. The sample size *n* = 369.

Additionally, the causal analysis indicated that daily step count had a statistically significant effect on average glucose. An increase in one unit, i.e., 1000 steps, led to an absolute decrease of 0.3 mg/dL (*p* = 0.02, linear regression *t*-test) in average glucose. However, the effect on time-in-range was not significant.

### Post-meal step count to reduce meal event’s glucose response and risk of elevated classification

We also investigated if the post-meal step count of a meal event could potentially be used as a targeted treatment to reduce the meal event’s glucose response and prevent an elevated classification. As our dataset was collected in free-living conditions, the size of the meal (calorie content) was not fixed. Hence, we adjusted for calorie content in our statistical analysis. To investigate the impact of post-meal step count on a meal event’s glucose response, we analyzed a subset of meal events that satisfied two conditions. First, the self-reported meal log (with non-zero calorie content) corresponding to the meal event should have high agreement in the corrected timing among the annotators. Specifically, the log was chosen if three out of the four corrected timings provided for that log were tagged in the same group (meal event) by the DBSCAN algorithm. Second, the post-meal step count was recorded during the meal event.

We computed ${\rm{MGR}}_{3\,{\rm{h}}}$ for each of 764 meal events and classified each of the meal events into elevated or normal categories using its ${\rm{MGR}}_{3\,{\rm{h}}}$. We found that post-meal step count had a significant effect on the meal event’s glucose response after adjusting for calorie content. An increase of 1000 steps leads to a decrease of ${\rm{MGR}}_{3\,{\rm{h}}}$ by 641 min mg/dL (*p* = 0.0006, linear regression *t*-test). An increase of 1000 steps in post-meal step count is associated with a 55% (*p* < 0.0001, linear regression *t*-test) decrease in the odds of elevated classification of the meal event. This finding suggests that post-meal physical activity can be used as an intervention to reduce the risk of elevated meal events and reduce meal events’ glucose response.

We demonstrated the effect of calorie content on the ${\rm{MGR}}_{3\,{\rm{h}}}$ and elevated/normal classification of the meal event after adjusting for post-meal step count. An increase in 100 kcal leads to an increase in ${\rm{MGR}}_{3\,{\rm{h}}}$ by 183 min mg/dL (*p* < 0.0001, linear regression *t*-test) and an 8% (*p* = 0.002, linear regression t-test) increase in the odds of elevated classification of the meal event.

## Discussion

Digital health represents the convergence of healthcare with technology (devices and software), such that wearable devices, information technology and communication tools come together to support people living with or at risk of developing diabetes. However, there is growing evidence that minority communities facing a disproportionate burden of diabetes and health disparities often lack access to currently available digital health technologies^18–20^. Our study demonstrated the feasibility and acceptability of multimodal digital phenotyping using digital health technologies in a U.S. Hispanic/Latino population with or at risk of T2D. Facilitating the use of digital health technologies for underserved communities may be one approach to help reduce the barriers to this underserved community participating in clinical research^21^.

In this study, we assessed the contribution of individual meals to a 3-h integrated area under the curve glucose responses in free-living conditions and demonstrated that an individual’s average meal event glucose response (across all their meals) did appear to be predictive of the HbA_1c_ level. It could be used to monitor T2D progression as well as provide a more personalized target for pharmaceutical and non-pharmaceutical interventions. Previously, in a similar population of Hispanic/Latino adults living with or at risk of developing T2D, it was possible to also predict HbA_1c_ from the breakfast glucose response alone^14^. In that study, self-reported meal logs were not collected and only breakfast responses were studied because it was easiest to annotate manually.

Current CGM metrics such as time-in-range characterize behavior across days so targets in time-in-range cannot be easily translated to event-driven recommendations. Biomarkers such as glycemic index that can be used to inform meal events are not suitable for free-living meal events that consist of multiple food items consumed together, often followed by physical activity^16,22,23^. In this study, to characterize meal events, we leveraged the function mapping average meal event glucose response to HbA_1c_ for classifying each individual meal event into a simple “normal” or “elevated” glucose response category. We found that an increase in the elevated meal event count was associated with a significant lowering of daily time-in-range between 54 and 140 mg/dL and an increase in average glucose after adjusting for covariates such as physical activity. Increasing the elevated meal event count of two or more each day had a significant effect on daily time-in-range and average glucose. As a corollary, any effect on daily glycemic outcomes due to an increase in elevated meal event count from zero to one was insignificant. Generally, 70 mg/dL is a lower limit for type 2 diabetic individuals. For completeness, we also performed our analysis with a lower limit of 70 mg/dL and found consistent results. Our results with a lower limit of 70 mg/dL are presented in Supplementary Table 12. This finding suggests that, at an individual level, one elevated meal event may be acceptable, while two or more elevated meal events significantly increase the potential for progression of dysglycemia. Daily elevated meal event count from CGM and meal logging may potentially be used as a glycemic target parameter by healthcare providers. The binary characterization of a meal event from CGM and diet logging could be used to provide personalized CGM-driven food reflection reports similar to calorie density color codes proposed in the prior work^24^.

Participants also recorded their daily levels of physical activity in this study. For people living with T2D, physical activity is encouraged as part of the management of glycemia and overall health based on evidence that regular physical activity can help to improve glycemic control as measured by changes in HbA_1c_ levels and 24-h ambulatory glucose profiles^25,26^. In addition, reducing the amount of sedentary time may also help prevent T2D for those at risk^27^. In this study, an increase in physical activity (represented as step count increase) within one hour after a meal event was associated with a significantly lower meal event glucose response and risk of an elevated classification. Previously^15^ we reported that, for free-living Hispanic/Latino adults with or at risk of T2D, there appears to be clustering of their daily levels of physical activity by intensity and time of day, which, in turn, may influence achieved HbA_1c_ and BMI. In that study, the amount of physical activity was more impactful on the HbA1c achieved among participants who were more active later during the day, as well as for overweight and younger individuals^15^. Taken together with this study, these findings suggest that targeted step count recommendations could be provided as a just-in-time adaptive intervention^28^. For example, after an individual logs their meal, a post-meal step count needed to prevent an elevated meal event is recommended. Another example is that if a low post-meal step count is detected after the meal, a notification encouraging you to take a walk is sent. Further, if an elevated meal event is detected, a notification to engage in some form of physical activity after subsequent meals can be provided.

The study has two limitations. First, the participants self-reported their meals in the MyFitnessPal App. While self-reported food diary data may be prone to inaccuracy, recent research analyzed large datasets collected through MyFitnessPal to derive insights about dietary behaviors^29–31^. In our study, we observed that participants often logged their meals later in the day. Hence, the self-reported data is prone to inaccuracy, especially when more time has passed between the actual meal and entry into the app. We leveraged the synchronous CGM curves and prior knowledge that meals lead to a sharp increase in glucose values to combat the inaccuracies in self-reported timing. In our analysis, we implemented a meal timing correction and addition framework manually. However, manual annotations were subject to human error. To reduce bias, we aggregated annotations across four independent annotators. In the future, automated meal annotation tools can substitute for manual annotations. Although automated meal annotation tools have been developed for type 1 diabetes^32^, they need to be validated on datasets comprising of individuals with or at risk of T2D. In real-world deployment scenarios, a real-time meal detection tool could be used to remind participants to log or take a photo of their meals and provide targeted just-in-time recommendations^33^.

The second limitation is that the generalizability of the study is limited due to the small sample size (36 participants). The error of 0.8% is large, considering the HbA_1c_ statistics in our dataset is 6.0 [IQR: 5.5–6.9]. However, we do show that most of the error arises due to the model’s inability to accurately fit for high HbA_1c_ (HbA_1c_ > 10) values mainly because of the low representation of such high HbA_1c_ values in our dataset. Excluding the four high HbA_1c_ samples, the error reduces to 0.41%. We also tried a quadratic term fit shown in Supplementary Fig. 1, and the error was reduced to 0.75% and 0.4% (excluding the HbA_1c_ > 10% samples). The trends in our results remain unchanged, as shown in Supplementary Table 11. The model mapping average meal event glucose response to HbA_1c_ is a population model and would be more accurate with a larger sample size and more balanced representation across HbA_1c_ values. Causal effects estimated on observational data are a function of the population. Population behaviors may be widely different for other ethnic and racial groups, especially regarding diet and physical activity^34,35^. Hence, the effects need to be validated with a larger sample size and compared to other populations with different ethnicities. Given that the U.S. Hispanic/Latino population faces a disproportionate burden of diabetes compared to the background population^11^, elucidating the association between diet, physical activity, and glycemic control has been the focus of our work. Our study suggests that the use of digital health technologies is both feasible and acceptable for this population and could potentially help achieve more equitable health outcomes^36^.

In conclusion, we conducted a multimodal digital phenotyping study to investigate the impact of meal events and physical activity on daily glycemic control in Hispanic/Latino adults with or at risk of T2D. We proposed a CGM-driven binary characterization of a meal event that is the elevated/normal classification of a meal event by exploiting the association found between participants’ average meal event glucose response and HbA_1c_. We introduced the concept of an elevated meal event count and showed that it significantly contributed to time-in-range between 54 and 140 mg/dl and average glucose. In this study, we also found that the post-meal step count significantly impacts the risk of elevated meal events. A specific focus of the application of digital phenotyping is the ability to visualize and interpret physiological data from digital health technologies that can potentially create opportunities for new therapeutic interventions at an increasingly personal level, benefiting the majority by focusing on the uniqueness of individuals. The findings of our study may offer the opportunity for non-pharmacologic interventions for populations facing a disproportionate burden of T2D.

## Methods

### Study

Thirty-six adult participants of self-reported Hispanic/Latino heritage with a diagnosis of T2D or at risk for developing T2D using the American Diabetes Association diabetes risk assessment tool^37^ provided written informed consent to be enrolled in a prospective, observational cohort study (ClinicalTrials.gov number: NCT04820348). The American Diabetes Association created a simple 7-question test to estimate someone’s risk of having diabetes. The test considers age, gender, history of gestational diabetes, family history of diabetes, high blood pressure, level of physical activity, and BMI. Each question is scored from 0 to 1 or more points. A total score of 5 or higher indicates the person is at high risk for having diabetes. The goal of the test is to identify those with modifiable risk factors and raise awareness so they can make lifestyle changes to prevent diabetes.

Baseline measurement of HbA_1c_ was taken using point-of-care HbA_1c_ testing (Alere Afinion 2). Multimodal monitoring involves three digital health modalities: continuous glucose monitoring (CGM), a diet-tracking mobile application, and physical activity monitoring. Participants wore a blinded CGM (Abbott Freestyle Libre Pro) for 14 days after enrollment. During enrollment, the premium version of the MyFitnessPal (MyFitnessPal, Inc.) app was installed on each participant’s personal smartphone in the desired language (English/Spanish). Study staff trained the participants to use the MyFitnessPal app. Participants reported the timing; meal occasion (breakfast, lunch, dinner, or snacks); name; and quantity of the food item by searching the MyFitnessPal food database. Based on the quantity selected and available information about the food item in the database, the app calculated the macronutrient composition for each food item. For measurement of physical activity, the participants wore the ActiGraph wGT3X-BT (ActiGraph, Pensacola, Florida, USA) on their dominant hip with an elastic belt for 24 hours/day except when bathing, swimming, and sleeping for 14 days. The downloaded data were screened for wear time using the Choi algorithm based on previous work using the manufacturer’s software (ActiLife 6.13.3)^38^. Participants were asked to continue normal activities during the study period. The adherence measure of MyFitnessPal meal logging was the number of days the participant logged food for two or more different meals^39^. The adherence measure of the CGM device was the number of days with no missing glucose values. ActiGraph adherence was the number of days 10 or more hours of step counts were recorded^40^. Past work^41^ has established that within 14 days, having at least 10 days (70%) of CGM data is required to measure usual glycemic patterns reliably. Hence, this study considers ten days of simultaneous adherence to MyFitnessPal, ActiGraph, and CGM. Simultaneous adherence days refer to days with no missing values of CGM, ActiGraph recorded ten or more hours of step counts, and at least two meals were logged on MyFitnessPal.

### Ethics approval

Ethics approval was obtained from the Rice University Institutional Review Board (IRB-FY2021-54). Written informed consent was obtained from all participants.

### Segmentation of meal events

We used the self-reported meal log timings and CGM curves to identify the start time of meal events. We segmented 3 h of the CGM curve from the start time as the meal event. Participants were asked to log their meals just before eating so that the meal log timing would align with the sharp rise in glucose levels seen in the CGM curve. However, after the study was completed, it became apparent that the meals were often logged variably after eating, with the meal log timing not always coinciding with the rise in glucose levels. Also, in some instances, participants forgot to log their meals. Hence, we implemented a framework to correct the meal log timings and add meal timings.

### Framework to correct and manually add meal log timings

While analyzing meal logs, we observed that participants entered food items consumed in a given meal in stages. For example, under breakfast for an example participant, oatmeal was logged at 10 am, and orange juice at 10:15 am. We combined food logs with the same meal occasion label (e.g., breakfast) and timing difference within 60 minutes into a single meal event. The meal log timing assigned to the meal event was the timing of the first food log in that meal (10 am in the example above).

For each day, we overlaid the meal log timings on the 24-h CGM curve. Based on visual inspection, the starting time point of a bell-shaped CGM curve segment (between 4 am and 11:59 pm) with sharp glucose rise closest to the meal log timing with minimal overlap with other 3-h CGM curve segments corresponding to other meal logs was selected as the corrected timing. In Fig. 3, the orange boxes highlight examples where the meal log timing is delayed with respect to the start of a sharp rise in glucose levels, and the green lines denote the corrected timing. The time difference between the corrected timing and self-report timing varies as seen in the time difference between the green and red lines in the orange boxes in Fig. 3a, c, d. The green boxes highlight examples on the same day where the meal log timing coincided perfectly with the sharp rise in glucose rise.

In some cases, participants logged multiple meal events with different meal occasion labels at the same time. For example, when a participant logged all the meals at the end of the day. In such scenarios, multiple bell-shaped CGM segments with a sharp glucose rise were visually identified for each meal event. The meal event timings were aligned in the order specified by the meal occasion label, breakfast followed by lunch, followed by dinner. Snacks were logged at the same time as other meals and tagged unknown. Meal logs for which a corrected timing could not be identified were tagged unknown.

In cases where a timing in the self-reported log was missing or none of the meal events of the day were self-reported, we added the timings. Since we did not have the self-reported meal log timing as a reference, we visually identified bell-shaped CGM curve segments (between 4 am and 11:59 pm) with a sharp glucose rise. An example in which all meals were added is displayed in Fig. 3b. Another example in which only one of the meal events was added is highlighted in Fig. 3d (yellow line).

### Aggregation of annotated timings for identification of the start time of meal event

For each day of each participant, we used Density-based spatial clustering of applications with noise (DBSCAN)^42^ algorithm to aggregate the annotated timings into groups. The Sklearn package in Python was used to implement DBSCAN with parameters ${\rm{eps}}\,=\,1.5$ and ${\rm{minsamples}}\,=\,2$. The parameters were chosen such that each group had at least 2 annotated timings and groups were at least 1.5 h apart. Annotated timings that were tagged as outliers by the algorithm were removed. Each group symbolized a meal event if the start time of the group (i.e., the annotated timing that had the largest incremental area under the 3-h CGM curve) had a maximum glucose rise ≥18.6 mg/dL. The threshold of 18.6 mg/dL was based on a recent report that 97.5% of post-meal glucose profiles in healthy adults have a maximum glucose rise greater than 18.6 mg/dL^43^.

### Elevated/normal classification of meal event

We developed a new CGM-driven meal event characterization that uses an “elevated” or “normal” classification of the meal. For each meal event, we computed incremental area under the 3-h CGM curve denoted as ${\rm{MGR}}_{3\,{\rm{h}}}$ using: (i) the 3-h CGM curve segment beginning from the start time of the meal event, (ii) the baseline glucose value at the the start time of the meal event is subtracted from the glucose values in the 3-h segment, and (iii) negative glucose values set to zero. The trapezoidal rule is used to calculate the area under the curve. The incremental area under the curve is widely used to characterize glucose responses to meals in previous works^14,16^. A characterization of meal events based on ${\rm{MGR}}_{3\,{\rm{h}}}$ does not exist for individuals with or at-risk of diabetes. However, ${\rm{HbA}_{1c}}$ thresholds for prediabetes (5.7–6.4%) and type 2 diabetes (>6.4%) are well established in the literature. Hence, we developed a new CGM-driven meal event characterization that uses an “elevated” or “normal” classification of the meal by exploiting the mapping between ${\rm{MGR}}_{3\,{\rm{h}}}$ and ${\rm{HbA}_{1c}}$ observed across participants (explained in “Methods”).

For every participant, we calculated their average meal events’ glucose response denoted as ${\overline{\rm{MGR}}_{3\,{\rm{h}}}}$. By aggregating meal events’ glucose responses, variations due to differences in food choices and physical activity around meals were smoothed out and the association with HbA_1c,_ which is also an average metric, was more prominent.

We built a robust linear regression model^44^ with ${\rm{HbA}_{1c}}$ as response variable and ${\overline{\rm{MGR}}_{3\,{\rm{h}}}}$ as a predictor variable. The robust linear regression model was denoted as ${\rm{HbA}_{1c}}={f}({\overline{\rm{MGR}}_{3\,{\rm{h}}}})+\epsilon$ where $\epsilon \in {N}(0,{\sigma }^{2})$ and$\,f$ is a linear function. We used the learned function to characterize individual meal events as elevated or normal (from a glucose perspective). The model is utilized to find a hypothetical HbA_1c_ mapping for a single meal’s ${\rm{MGR}}_{3\,{\rm{h}}}$. The HbA_1c_ values of a single meal signify the hypothetical scenario where if the individual ate that meal all the time and their average meal event glucose response was equal to that meal event’s glucose response, what would be their HbA_1c_? We use this hypothetical HbA_1c_ to classify the meal event as normal or elevated.

We define elevated meal events differently for at-risk, prediabetes, and T2D participants. Thus, for at risk and prediabetes participants, the classification of the meal event’s glucose response was elevated if $f({\rm{MGR}}_{3\,{\rm{h}}})$ ≥ 5.7 and normal if $f({\rm{MGR}}_{3\,{\rm{h}}})$ < 5.7. In the case of type 2 diabetes participants, the meal event’s glucose response was elevated if $f({\rm{MGR}}_{3\,{\rm{h}}})$ ≥ 6.5 and normal if $f({\rm{MGR}}_{3\,{\rm{h}}})$ < 6.5. With the above formulation, we proposed a new CGM-driven meal event characterization that can be used to classify all detected meal events into elevated versus normal categories. For example, consider a participant with prediabetes consumed a meal event with a mapped HbA_1c_ of 6.0. A mapped HbA_1c_ of 6.0 indicates that the particular meal event’s glucose response is similar to the average meal event’s glucose response of a participant with T2D. Then repeated consumption of that meal event is unsafe for the prediabetic participant and so the meal event receives an elevated classification for the prediabetic participant.

### Meal event biomarkers

#### Meal event’s glucose response

We defined a meal event’s glucose response as the incremental area under the 3-h CGM curve from the start time of the meal event and denoted it as ${\rm{MGR}}_{3\,{\rm{h}}}$.

#### Post-meal step count of the meal event

We represented the post-meal step count of the meal event as the step count in the 1-h time window after the start time of the meal event. We selected a one-hour window based on previous works that showed that physical activity 15–30 min after completion of the meal reduced postprandial glucose responses^45,46^.

#### Calorie content of the meal event

This was calculated as the sum of the calorie content of individual food items in the self-reported meal log corresponding to the particular meal event.

#### Elevated/normal classification of meal event

Classification of the meal event based on its ${\rm{MGR}}_{3\,{\rm{h}}}$ was into normal or elevated categories. We describe the elevated/normal classification of meal events in detail below.

### Daily biomarkers

#### Duration of the day

The duration of the day was defined as the time period between the start time and end time of our analysis window for that day. Our analysis focuses on the effect of meals and physical activity. Hence, the start time of our analysis window of a day is the earlier time between the start time of the first meal and the earliest detected ActiGraph wear time after 4 AM. The end time is later, between 3 hours after the start time of the last meal event and the last detected ActiGraph wear time before 11:59 PM. We consider the start time of the last meal event so that we include the physiological response of the meal event in our analysis window. The duration of the day differs across days and individuals.

#### Elevated meal event count of the day

The elevated meal event count of a day summarizes the number of meal events that were classified as elevated (defined in the previous section) on that day. Elevated meal event count was defined as a multi-category variable with four categories: zero elevated meal event, one elevated meal event, two elevated meal events, and ≥3 elevated meal events.

#### Daily step count of the day

The step count of the day was the total steps taken during the duration of the day.

#### Baseline glucose of the day

The baseline glucose of the day was defined as the glucose value in the CGM curve at the start time of the day.

#### Glycemic outcomes of the day

The daily glycemic outcomes considered were time-in-range (54–140 mg/dL), and average glucose was measured using CGM values during the duration of the day. Time-in-range (TIR_54__–__140_) was calculated as the percentage of time the glucose measurements were within 54–140 mg/dl. A lower limit of 54 mg/dl was chosen because at-risk and prediabetic individuals can have non-pathologic glucose values below 70 mg/dL^47^. Further, the range of <54 mg/dL is defined as clinically significant hypoglycemia in nondiabetic individuals^48^. An upper limit of 140 mg/dL was chosen based on recent findings that daytime time-in-range of 140–180 mg/dl can be used as an early indicator of diabetes progression^14^.

We computed elevated meal event count, daily step count, baseline glucose, and duration for each day and participant. For daily measures, we excluded days where no meals were identified, the ActiGraph wear time for that day was less than 10 h (between 4 am and 11:59 pm), or the CGM data was incomplete (the first and last day of the study). Additionally, we removed days where the CGM profile showed measurements ≤40 mg/dL and/or ≥500 mg/dL (glucose values were beyond the sensor limits of the CGM device).

### Causal analysis

In real-world datasets, variables are often correlated with each other. Biology and behaviors of an individual have a complex interplay as shown in Fig. 1c. Our computed biomarkers are expected to be associated with each other. Hence to isolate the causal impact of a variable of interest, we need to account for other variables as confounding covariates and adjust for them in our analysis.

Propensity score models are required to estimate the causal effect of a variable of interest on outcome variables from observational data. The covariate variables need to be balanced if the variable of interest and covariates are correlated to ensure there is no confounding. We used inverse probability weighting, a widely used propensity score weighting estimator for balancing the covariates and measuring the causal effects^49^. The generalized propensity score for a sample $i$, for a variable of interest $T,$ and confounding covariates $X$ is given as ${e}_{i}={P}({T}_{i}={t}/{X}_{i})$ and is calculated using a propensity score model. The inverse probability weights are given as ${w}_{i}=1/P({T}_{i}={t}/{X}_{i})$. The inverse probability weights are then used as weights in a weighted regression between the variable of interest $T$ and outcome variable $Y$. When the covariate balance is not completely achieved with the inverse probability weights, doubly robust approach that is fitting the weighted outcome regression with both $T$ and $X$ as input variables are performed^50^. We used the estimated coefficients of the outcome regression to quantify the effect. We also report the statistical significance (*p*-value) of the estimated coefficients.

We estimated four causal effects in our analysis. First, the causal effect of elevated meal event counts on glycemic outcomes. Second, the causal effect of daily step count on glycemic outcomes. Third, the effect of post-meal step count on glycemic response of meal events. Lastly, the effect of calorie content on the glycemic response of meal events.

As our dataset consists of multiple data samples from multiple participants, we use multilevel regression models in our causal analysis. The multilevel structure was captured using the user ID. For continuous biomarkers such as daily step count, post-meal step count and calorie content, we used linear mixed effect models as the propensity score model and normal density to transform estimates into probability scale weights^51^. For multi-category biomarkers such as the elevated meal event count, we used the multinomial mixed effects model as the propensity score model. We assessed the covariate balance before and after inverse probability weighting using weighted correlations between variable of interest and covariates for continuous treatments^52^. An absolute correlation coefficient of less than 0.1 is desired to assume covariate balance^52^. For multi-category treatments, we calculated the maximum standardized mean difference between all pairwise treatment group comparisons. A difference of less than 0.2 is desired for covariate balance^53^.

To compute the effect of elevated meal event count on glycemic outcomes, we considered the elevated meal event count as the variable of interest, with covariates being baseline glucose level, duration of the day, and daily step count and outcomes being TIR_54__–__140_ and average glucose. The regression model is summarized in Supplementary Tables 3 and 4, and the covariate balance is achieved in Supplementary Table 2. While computing the effect of daily step count, the covariates were baseline glucose level, elevated meal event count, and duration of the day. Supplementary Tables 6 and 7 summarize the regression model, and the covariate balance is displayed in Supplementary Table 5.

Next, we considered post-meal step count as the variable of interest, the calorie content of the meal as the covariate and ${\rm{MGR}}_{3\,{\rm{h}}}$ and likelihood of elevated meal event classification as the outcome variables. Inverse probability weighting was not implemented as the correlation between the post-meal step count and the calorie content was −0.05, which was low enough to assume adequate covariate balance. Thus, we did multiple regression using a linear mixed effects model to estimate the effect of post-meal step count and calorie content on ${\rm{MGR}}_{3\,{\rm{h}}}$ (Supplementary Table 8). We used a generalized mixed effects model to compute the effect of post-meal step count and calorie content on elevated/normal classification of the meal event (Supplementary Table 9).

Custom code was developed for statistical analyses in Python (version 3.9.13) and R (version 4.2.0). The RLM function from the statsmodel package in Python was used for robust linear regression. The linear mixed-effect regression was performed using the lmer function from lmerTest library in R. The mblogit function from mclogit library in R was used for multinomial mixed-effect regression. The covariate balance was assessed with the cobalt package in R^54^. All custom code written as a Jupyter notebook is available on request. Statistical significance is defined as a *p*-value < 0.01.

### Reporting summary

Further information on research design is available in the Nature Research Reporting Summary linked to this article.

### Supplementary information

Supplementary

Reporting Summary

## Data Availability

Data are available upon reasonable request. The investigators agree to share de-identified individual participant data that underlie the results reported in this article and the study protocol. Requests should be directed to wbevier@sansum.org. To gain access, data requestors will need to sign a data access agreement.

## Code availability

The computational and statistical analysis tools developed for this study are available upon request. Requests should be directed to the corresponding author.
