# Supplementary material for: Multimodal digital phenotyping of diet, physical activity, and glycemia in Hispanic/Latino adults with or at risk of type 2 diabetes
Source: NPJ Digit Med. 2024 Jan 11;7:7. doi: 10.1038/s41746-023-00985-7 (PMC10784546; doi:10.1038/s41746-023-00985-7)
Supplement: Supplementary file 1 — Supplementary [file 41746_2023_985_MOESM1_ESM.pdf]

# Supplementary Material

**Supplementary Table 1:** Annotation details.

| Annotator | Number of logs for which corrected timing was identified | Number of manual timings added |
|---|---|---|
| 1 | 1154 | 601 |
| 2 | 1078 | 630 |
| 3 | 1064 | 444 |
| 4 | 1356 | 398 |

**Supplementary Table 2:** Covariate balance achieved between elevated meal event count and covariates after inverse probability weighting.

| Balance measures between elevated meal event count and covariates | Type of Variable | Maximum standardized mean difference across all pairwise comparisons | |
|---|---|---|---|
| | | Before inverse probability weighting | After inverse probability weighting |
| Baseline glucose | Continuous | 0.57 | 0.41 |
| Step counts | Continuous | 0.32 | 0.25 |
| Duration | Continuous | 0.39 | 0.11 |

**Supplementary Table 3:** Output of linear mixed effects model to estimate effect of elevated meal event count on time-in-range (54-140 mg/dL).

| | | Estimate | Standard Error | P-value (t-test) |
|---|---|---|---|---|
| Intercept | | 99.4 | 6.7 | <0.0001 |
| Elevated meal event count | 0 to 1 | -1.1 | 1.3 | 0.41 |
| | 0 to 2 | -4.0 | 1.3 | 0.003 |
| | 0 to ≥3 | -8.1 | 1.5 | <0.0001 |

| | | | |
|---|---|---|---|
| Daily step counts | -0.2 | 0.2 | 0.30 |
| Baseline glucose | -0.3 | 0.02 | <0.0001 |
| Duration | 0.6 | 0.3 | 0.03 |

**Supplementary Table 4:** Output of linear mixed effects model to estimate effect of elevated meal event count on average glucose.

| | | Estimate | Standard Error | P-value (t-test) |
|---|---|---|---|---|
| Intercept | | 92.5 | 7.6 | <0.0001 |
| Elevated meal event count | 0 to 1 | 1.7 | 1.2 | 0.16 |
| | 0 to 2 | 5.8 | 1.3 | <0.0001 |
| | 0 to ≥3 | 7.7 | 1.4 | <0.0001 |
| Step counts | | -0.3 | 0.2 | 0.08 |
| Baseline glucose | | 0.3 | 0.02 | <0.0001 |
| Duration | | -0.5 | 0.3 | 0.05 |

**Supplementary Table 5:** Covariate balance achieved between daily step counts and covariates after inverse probability weighting.

| Balance measures between step counts and covariates | | Type of Variable | Correlation | |
|---|---|---|---|---|
| | | | Before inverse probability weighting | After inverse probability weighting |
| Baseline glucose | | Continuous | -0.19 | -0.18 |
| Elevated meal event count | 0 | Binary | 0.08 | 0.03 |
| | 1 | Binary | -0.08 | -0.05 |

| | | | | |
|---|---|---|---|---|
| | 2 | Binary | 0.02 | 0.03 |
| | 3 | Binary | -0.02 | -0.0 |
| Duration | | Continuous | 0.15 | 0.09 |

**Supplementary Table 6:** Output of linear mixed effects model to estimate effect of daily step count on time-in-range (54-140 mg/dL).

| Fixed effects | | Estimate | Standard Error | P-value (t-test) |
|---|---|---|---|---|
| Intercept | | 100.0 | 6.5 | <0.0001 |
| Step counts | | 0.07 | 0.1 | 0.63 |
| Elevated meal event count | 0 to 1 | -1.3 | 1.5 | 0.40 |
| | 0 to 2 | -3.8 | 1.5 | 0.01 |
| | 0 to ≥3 | -7.7 | 1.9 | <0.0001 |
| Baseline glucose | | -0.3 | 0.02 | <0.0001 |
| Duration | | 0.4 | 0.3 | 0.13 |

**Supplementary Table 7:** Output of linear mixed effects model to estimate effect of daily step count on average glucose.

| Fixed effects | | Estimate | Standard Error | P-value (t-test) |
|---|---|---|---|---|
| Intercept | | 91.8 | 7.8 | <0.0001 |
| Step counts | | -0.3 | 0.1 | 0.02 |
| Elevated meal event count | 0 to 1 | 2.5 | 1.4 | 0.08 |
| | 0 to 2 | 5.7 | 1.4 | <0.0001 |

| | | | | |
|---|---|---|---|---|
| | 0 to ≥3 | 7.6 | 1.7 | <0.0001 |
| Baseline glucose | | 0.3 | 0.02 | <0.0001 |
| Duration | | -0.4 | 0.2 | 0.09 |

**Supplementary Table 8:** Output of linear mixed effects model to estimate effect of post-meal step count on meal event glucose response.

| Fixed effects | Estimate | Standard Error | P-value (t-test) |
|---|---|---|---|
| Intercept | 4755.0 | 436.1 | <0.0001 |
| Post-meal step count | -640.6 | 185.4 | 0.0006 |
| Calorie content | 182.8 | 33.0 | <0.0001 |

**Supplementary Table 9:** Output of linear mixed effects model to estimate effect of post-meal step count on elevated classification of meal event.

| Fixed effects | Estimate | Standard Error | P-value (t-test) |
|---|---|---|---|
| Intercept | -0.2 | 0.2 | 0.43 |
| Post-meal step count | -0.8 | 0.2 | <0.0001 |
| Calorie content | 0.08 | 0.03 | 0.002 |

**Supplementary Table 10:** Occupation information of the participants.

| Occupation | Number of participants |
|---|---|
| Promoter | 3 |
| Counselor | 1 |

| | |
|---|---|
| Substitute teacher | 1 |
| Retired | 2 |
| Student | 1 |
| Deli | 1 |
| Records technician | 1 |
| Cleaning service | 1 |
| Housewife | 2 |
| Gardener | 1 |
| Service Counter Representative (Bank) | 1 |
| Housekeeping | 9 |
| Housemaid | 1 |
| Nurse | 1 |
| Housewife/student | 1 |
| Customer service | 1 |
| Cook | 2 |
| Cashier | 1 |
| None | 1 |
| Guest representative | 1 |
| Clerk | 1 |
| Engineer | 1 |
| Full service | 1 |

**Supplementary Table 11:** Main results obtained with quadratic term fit between average meal event glucose response and HbA1c. Effect of daily step count and elevated meal event count on time-in-range (54-140 mg/dL) and average glucose. Effect of post-meal step count and calorie content on meal event glucose response and elevated classification of meal event.

| Outcome | | Time-in-range (54-140 mg/dL) Percentage of total time (%) | | | Average glucose mg/dL | | |
|---|---|---|---|---|---|---|---|
| Variable of interest | | Estimate | Standard Error | P-value (t-test) | Estimate | Standard Error | P-value (t-test) |
| Elevated meal event count | 0 to 1 | -0.4 | 1.3 | 0.7 | 1.4 | 1.2 | 0.26 |
| | 0 to 2 | -4.0 | 1.3 | 0.003 | 5.8 | 1.3 | <0.0001 |

| | | | | | | | |
|---|---|---|---|---|---|---|---|
| | 0 to ≥3 | -6.9 | 1.5 | <0.0001 | 7.3 | 1.5 | <0.0001 |
| Daily step count (1 unit = 1000 steps) | | 0.08 | 0.2 | 0.58 | -0.4 | 0.1 | 0.009 |
| Outcome | | Meal event glucose response | | | Elevated classification of meal event | | |
| Variable of interest | | Estimate | Standard Error | P-value (t-test) | Estimate | Standard Error | P-value (t-test) |
| Post-meal step count (1 unit = 1000 steps) | | -640.0 | 185.4 | 0.0006 | -0.8 | 0.2 | <0.0001 |
| Calorie content ( 1 unit = 100 kcal) | | 182 | 34.0 | <0.0001 | 0.1 | 0.03 | 0.0005 |

**Supplementary Table 12:** Effect of elevated meal event count on time-in-range (70-140 mg/dL).

| Outcome | | Time-in-range (70-140 mg/dL) Percentage of total time (%) | | |
|---|---|---|---|---|
| Variable of interest | | Estimate | Standard Error | P-value (t-test) |
| Elevated meal event count | 0 to 1 | -1.4 | 1.4 | 0.35 |
| | 0 to 2 | -4.4 | 1.5 | 0.003 |
| | 0 to ≥3 | -8.4 | 1.6 | <0.0001 |

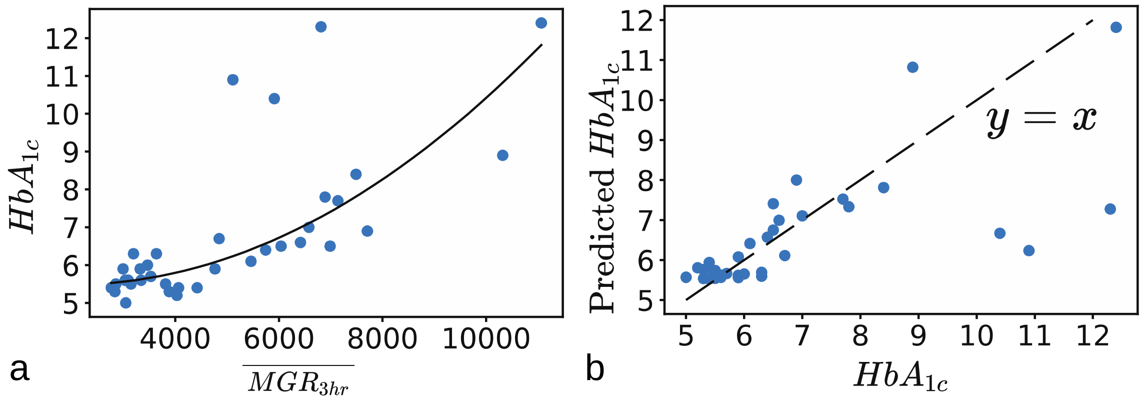


**Supplementary Figure 1:** Quadratic term mapping between average meal event glucose response and $HbA_{1c}$. The scatterplot (a) displays the robust regression fit between average meal event glucose response and $HbA_{1c}$. The

equation representing the best line-of-fit is reported (solid black line). The scatterplot in (b) shows the correlation between true and fitted $HbA_{1c}$ values. The dashed black line represents the 45-degree line.
