# Supplementary material for: Multimodal digital phenotyping of diet, physical activity, and glycemia in Hispanic/Latino adults with or at risk of type 2 diabetes
Source: NPJ Digit Med. 2024 Jan 11;7:7. doi: 10.1038/s41746-023-00985-7 (PMC10784546; doi:10.1038/s41746-023-00985-7)
Supplement: Supplementary file 2 — Reporting Summary [file 41746_2023_985_MOESM2_ESM.pdf]

nature portfolio

Corresponding author(s): DBPR NPJDIGITALMED-06627

Last updated by author(s): Sep 17, 2023

Reporting Summary

Nature Portfolio wishes to improve the reproducibility of the work that we publish. This form provides structure for consistency and transparency in reporting. For further information on Nature Portfolio policies, see our Editorial Policies and the Editorial Policy Checklist.

Statistics

For all statistical analyses, confirm that the following items are present in the figure legend, table legend, main text, or Methods section.

n/a	Confirmed	
☐	☒	The exact sample size (*n*) for each experimental group/condition, given as a discrete number and unit of measurement
☐	☒	A statement on whether measurements were taken from distinct samples or whether the same sample was measured repeatedly
☒	☐	The statistical test(s) used AND whether they are one- or two-sided<br>*Only common tests should be described solely by name; describe more complex techniques in the Methods section.*
☐	☒	A description of all covariates tested
☒	☐	A description of any assumptions or corrections, such as tests of normality and adjustment for multiple comparisons
☐	☒	A full description of the statistical parameters including central tendency (e.g. means) or other basic estimates (e.g. regression coefficient) AND variation (e.g. standard deviation) or associated estimates of uncertainty (e.g. confidence intervals)
☒	☐	For null hypothesis testing, the test statistic (e.g. *F*, *t*, *r*) with confidence intervals, effect sizes, degrees of freedom and *P* value noted<br>*Give P values as exact values whenever suitable.*
☒	☐	For Bayesian analysis, information on the choice of priors and Markov chain Monte Carlo settings
☐	☒	For hierarchical and complex designs, identification of the appropriate level for tests and full reporting of outcomes
☐	☒	Estimates of effect sizes (e.g. Cohen's *d*, Pearson's *r*), indicating how they were calculated

Our web collection on statistics for biologists contains articles on many of the points above.

Software and code

Policy information about availability of computer code

Data collection	ActiLife 6.13.3 commercial software to download Actigraph data<br>Abbott Freestyle Libre Pro commercial software to download data from continuous glucose monitor<br>https://github.com/coddingtonbear/python-myfitnesspal to download MyFitnessPal data
Data analysis	Custom code was developed for statistical analyses in Python (version 3.9.13) and R (version 4.2.0). The code developed for this study is available upon request. Requests should be directed at amrutarice@gmail.com

For manuscripts utilizing custom algorithms or software that are central to the research but not yet described in published literature, software must be made available to editors and reviewers. We strongly encourage code deposition in a community repository (e.g. GitHub). See the Nature Portfolio guidelines for submitting code & software for further information.

Data

Policy information about availability of data

All manuscripts must include a data availability statement. This statement should provide the following information, where applicable:

- Accession codes, unique identifiers, or web links for publicly available datasets
- A description of any restrictions on data availability
- For clinical datasets or third party data, please ensure that the statement adheres to our policy

Data is available upon reasonable request. The investigators agree to share de-identified individual participant data that underlie the results reported in this article and the study protocol. Requests should be directed to wbevier@sansum.org. To gain access, data requestors will need to sign a data access agreement.

Research involving human participants, their data, or biological material

Policy information about studies with human participants or human data. See also policy information about sex, gender (identity/presentation), and sexual orientation and race, ethnicity and racism.

Reporting on sex and gender	Self-reported data on sex was collected and aggregate is included in the manuscript. There were 28 female and 8 male participants. Due to the small sample size, sex-specific analysis was beyond the scope of the manuscript.
Reporting on race, ethnicity, or other socially relevant groupings	All participants were self-reported Hispanic/Latino
Population characteristics	See above
Recruitment	Potential participants will be recruited via bilingual (Spanish and English) outreach materials. Flyers, brochures, and/or informational handouts may also be used for recruitment. A diabetes risk assessment tool sourced from the American Diabetes Association may be used for recruitment purposes. Authorized research personnel from Sansum Diabetes Research Institute (SDRI) may contact potential participants. Individuals expressing interest by responding to recruitment materials may contact SDRI staff. SDRI staff will pre-screen each participant and may schedule the initial screening and enrollment visit at the time most convenient for the participant. The sample is susceptible to self-selected bias as most participants are female. We acknowledge generalizability as a limitation in the paper.
Ethics oversight	Rice University

Note that full information on the approval of the study protocol must also be provided in the manuscript.

Field-specific reporting

Please select the one below that is the best fit for your research. If you are not sure, read the appropriate sections before making your selection.

☐ Life sciences ☒ Behavioural & social sciences ☐ Ecological, evolutionary & environmental sciences

For a reference copy of the document with all sections, see nature.com/documents/nr-reporting-summary-flat.pdf

Behavioural & social sciences study design

All studies must disclose on these points even when the disclosure is negative.

Study description	This is a observational study. The data used in this is paper is strictly quantitative data primarily from wearable devices (e.g continuous glucose monitors)
Research sample	Hispanic/Latino participants with or at risk of type 2 diabetes<br>Age: median 51 [interquartile range 39 - 59] years<br>Sex: 28 female, 8 male<br>HbA1c : 6.0 [5.5 - 6.9] %
Sampling strategy	No sample size calculation was conducted. The study was a pilot study with 36 participants
Data collection	Baseline measurement of HbA1c was taken using point-of-care HbA1c testing (Alere Afinion 2). Participants wore a blinded CGM (Abbott Freestyle Libre Pro) for 14 days after enrollment. During enrollment, the premium version of the MyFitnessPal (MyFitnessPal, Inc.) app was installed on each participant's personal smartphone in the desired language (English/Spanish). For measurement of physical activity, the participants wore the ActiGraph wGT3X-BT (ActiGraph, Pensacola, Florida, USA). Self-reported survey information was recorded on paper
Timing	The first participant was enrolled in April 2021 and the last participant was enrolled in August 2021

Data exclusions	No participants were excluded. Preprocessing before data analysis was conducted to account for missing data. For various analysis, certain data samples (days) were excluded based on specific criteria described in detail in the paper.
Non-participation	No participant dropped out
Randomization	Participants were not allocated into experimental groups

Reporting for specific materials, systems and methods

We require information from authors about some types of materials, experimental systems and methods used in many studies. Here, indicate whether each material, system or method listed is relevant to your study. If you are not sure if a list item applies to your research, read the appropriate section before selecting a response.

Materials & experimental systems

n/a	Involved in the study
☒	☐ Antibodies
☒	☐ Eukaryotic cell lines
☒	☐ Palaeontology and archaeology
☒	☐ Animals and other organisms
☐	☒ Clinical data
☒	☐ Dual use research of concern
☒	☐ Plants

Methods

n/a	Involved in the study
☒	☐ ChIP-seq
☒	☐ Flow cytometry
☒	☐ MRI-based neuroimaging

Clinical data

Policy information about clinical studies

All manuscripts should comply with the ICMJE guidelines for publication of clinical research and a completed CONSORT checklist must be included with all submissions.

Clinical trial registration	NCT04820348
Study protocol	The study protocol may be accessed by contacting the Rice University IRB office.
Data collection	Data collection was conducted at Sansum Diabetes Research Institute between April and August 2021.
Outcomes	Adherence to wearing continuous glucose monitoring (CGM) devices to measure glucose levels. [Time Frame: 2 weeks]<br>Unit of measure: Percent of time and percent of days CGM is active during the study period.
